# The Interplay of TLR-NFκB Signalling Pathway and Functional Immune-Related Enzymes in the Inflammatory Response of *Ciona robusta*

**DOI:** 10.3390/ani14152169

**Published:** 2024-07-25

**Authors:** Luca Bisanti, Claudia La Corte, Mariano Dara, Federica Bertini, Jacopo Vizioli, Maria Giovanna Parisi, Matteo Cammarata, Daniela Parrinello

**Affiliations:** 1 Marine Immunobiology Laboratory, Department of Earth and Marine Sciences, University of Palermo, 90128 Palermo, Italy; luca.bisanti@unipa.it (L.B.); claudia.lacorte@unipa.it (C.L.C.); federica.bertini02@unipa.it (F.B.);; 2 National Biodiversity Future Center (NBFC), 90133 Palermo, Italy; 3 Laboratoire Protéomique, Réponse Inflammatoire et Spectrométrie de Masse (Inserm U1192), Département de Biologie, Université de Lille, F-59000 Lille, France

**Keywords:** ascidian, LPS, inflammation, innate immunity, *Ciona robusta*

## Abstract

**Simple Summary:**

Ascidians (Tunicata) are a powerful model for studying the innate immune system. To better understand the dynamics of immune responses under bacterial challenge in *Ciona robusta*, we exposed ascidians to lipopolysaccharide (LPS) injection. Immunohistochemistry analysis on two components of the nuclear factor kappa B (NfκB) key signalling pathway, the Toll-like receptor 4 (TLR4) and NFκB, showed their over-expression on tissue of LPS-injected ascidians. Also, several enzymes related to immune responses were up-modulated following the LPS challenge. Our study suggests a broad and complex innate immune activation in the regulation of tunicate inflammatory responses.

**Abstract:**

The close phylogenetic relationship between ascidians (Tunicata) and vertebrates makes them a powerful model for studying the innate immune system. To better understand the nature and dynamics of immune responses and the mechanisms through which bacterial infections are detected and translated into inflammation in *Ciona robusta*, we applied an approach combining in vivo lipopolysaccharide (LPS) stimulation, immune-labelling techniques and functional enzymatic analyses. The immunohistochemistry showed that Toll-like receptor 4 (TLR4) and nuclear factor kappa B (NFκB) were expressed during the inflammatory pharynx response 4 h post-LPS, with the formation of nodules in pharynx vessel lumen. Also, the endothelium vessels were involved in the inflammatory response. Observations of histological sections from naive and buffer-inoculated ascidians confirmed an immuno-positive response. Enzyme immune parameters—which included the activity of phenoloxidase, glutathione peroxidase, lysozyme, alkaline phosphatase and esterase—showed up-modulation 4 h after LPS injection, confirming their participation during ascidian inflammatory response. These findings provide new insights into the mechanisms underlying the LPS-induced *C. robusta* response and suggest that a broad innate immune mechanism, as in vertebrates, is involved in the regulation of inflammatory responses. Further findings in this direction are needed to cover knowledge gaps regarding the organized set of molecular and cellular networks involved in universal immune interactions with pathogens.

## 1. Introduction

Tunicates (urochordates) are generally considered to be the phylogenetically closest living relatives of vertebrates [1,2]. These organisms form a heterogeneous group spread across different marine habitats and have varied lifestyles (colonial or solitary, benthic or pelagic). Among these, the tunicate *Ciona robusta* Hoshino & Tokioka, 1967, is a non-colonial ascidian that lives mainly in clusters fixed in natural and artificial substrates. *C. robusta* is widely considered a powerful model for studying embryological development and innate immunity, and it possesses a high degree of genetic complexity, with protein homologues involved in vertebrate immunity being described for this organism in recent decades [3,4,5].

Like other filter-feeding invertebrates, this species lives in environments full of infectious agents (e.g., viruses, bacteria and fungi) and is characterized by a pharynx that has respiratory and nutritional functions as well as serving as the main immune organ [4]. *C. robusta* have developed obligatory relationships with harmful agents from the microbiome that can activate defence reactions, and they possess the capacity for self/non-self-recognition [4]. One of the primary functions of the tunicate innate immune system is to recognize non-self molecule-specific patterns [4,5,6] named pathogen-associated molecular patterns (PAMPs), such as peptidoglycan, lipopolysaccharide (LPS) and mannan components of the microbial cell wall or nucleic acids. These PAMPs and others are recognized by pattern recognition receptors (PRRs), which are proteins including both soluble proteins, such as lectins, and Toll-like receptors (TLRs) [3,4,5,6,7].

Several TLR genes with “hybrid” functionality have been identified in the genus *Ciona* [7,8], reflecting mammalian TLRs, and are generally divided into two subfamilies that mainly recognize related PAMPs: TLR1, TLR2, TLR4 and TLR6 recognize lipids, while TLR3, TLR7, TLR8 and TLR9 recognize nucleic acids [9,10]. The binding of PAMPs to TLRs activates cellular signalling cascades through myeloid differentiation primary response 88 protein (MyD88), which in turn binds members of the IL-1R-associated kinase (IRAK) family, thus leading to the activation of nuclear factor kappa B (NFκB) [11,12,13,14,15]. NFκB proteins, in turn, lead to the expression of proinflammatory cytokines (e.g., tumour necrosis factor α and interferons) [16,17,18] and are stored/sequestered in the cytoplasm by members of the κB family [19,20,21,22].

Tunicates utilize phenoloxidase (PO) for melanin biosynthesis like other invertebrates, with the enzymatic activity participating in inflammatory processes, wound healing, sclerotization, pigmentation, and defence [4,23,24,25]. Ascidian haemocytes contain a proenzyme (proPO) which is activated by PO through the serine protease cascade, which is in turn activated by PRRs after their binding to PAMPs [23,26,27,28,29]. Cytotoxic radicals produced by inflammatory reactions, such as reactive oxygen species (ROS), can also lead to cell oxidative stress, causing damage to tissue. Antioxidants, which readily scavenge oxygen radicals, are critical enzymes involved in functions related to cell immunity and phagocytosis during pathogen infection [30,31]. These include glutathione peroxidase (GPx), catalase, superoxide dismutase and fluorescent proteins.

Lysozyme (LYS) is a non-specific bacteriolytic enzyme characteristic of several groups of organisms, ranging from bacteria to animals, both vertebrate and invertebrate [32]. In *C. robusta*, it corresponds to the primary and rapid defence against pathogen attacks with bactericidal hydrolytic activity, which hydrolyses the β-1,4 glycosidic bonds of the bacterial cell wall, destabilizing the membrane [33,34,35]. In addition, the activity and kinetic characteristics of several metabolic enzymes, closely linked to immunity, are essential for maintaining invertebrate homeostasis following inflammatory activation [36]. Alkaline phosphatase (ALP) and esterase (EST) are examples of enzymes involved in a wide range of processes involving synthesis and hydrolysis reactions, as well as in various catabolic pathways in invertebrates [37,38,39].

Although the gene expressions involved in the immune response in *C. robusta* are well known (e.g., TLR, NFκB and PO), much remains to be understood about the wide-ranging nature and dynamics of immune activities in this ascidian during LPS exposure in vivo. In the present study, using an in vivo LPS-injection strategy, immuno-labelling techniques on pharynx tissues and enzyme activity readout, we investigated the response of *C. robusta* to LPS challenge. The combined approach provided valuable additional indications about the involvement of the TLR-NFκB-dependent pathway during the activation of inflammatory response following LPS injection. Furthermore, the functional activities of PO, GPX, LYS, ALP and EST enzymes were analysed for the first time in this ascidian species in LPS-mediated inflammatory response. These new findings indicate that at least the response circuits considered here, relevant to vertebrate immunity, were already in place in the common ancestor of the protochordates and vertebrates, broadening current knowledge on immune interaction evolution with pathogen agents.

## 2. Materials and Methods

### 2.1. Ascidian Collection and Experimental Design

Thirty adult ascidians were collected from Palermo Harbour (Sicily, Italy), maintained in aquaria with filtered seawater at 18 °C and fed with a Coraliquid diet from Sera Heinsberg, Germany. In the experimental plan, 10 control animals not subjected to injection were randomly sampled from the tanks, and tissues were fixed or frozen at −30 °C until tissue analyses. Then, 10 randomly chosen organisms were inoculated with LPS of *Escherichia coli* or marine solution (MS), and 4 h post injection were immediately fixed or stored at −30 °C. LPS of *E. coli* (ATCC 25922 strain; Chrisope Technologies, Lake Charles, LA, USA) was resuspended in MS (12 mM CaCl_2_·6H_2_O, 11 mM KCl, 26 mM MgCl_2_·6H_2_O, 43 mM TRIS HCl, 0.4 M NaCl, pH 8.0). LPS (100 µg in 100 µL MS per animal) or MS was inoculated under the tunic [4,40,41].

### 2.2. Immunohistochemistry

Before carrying out the immunohistochemical analyses, the antibody specificities against the selected target proteins in ascidian tissues were determined by checking the alignments between the deposited sequences of *C. robusta* (National Center for Biotechnology Information and UniProt databases) and the relative antibody epitopes (Figure S1; Table S1).

Body wall fragments of ascidians containing a pharynx alone or both a tunic and pharynx were excised at the injection site. Tissues were fixed in 4% paraformaldehyde in PBS-buffer solution (NaCl 137 mM, KH_2_HPO_4_ 10 mM, KH_2_HPO_4_ 2 mM, KCl 2.7 mM, pH 7.6) at 4 °C for 24 h. After dehydration in ethanol, animals were embedded in paraffin (Bio-Optica, Milan, Italy). Histological sections (7 μm thickness) were cut with a rotary automatic microtome (Leica Microsystems HM350S, Wetzlar, Germany). Immunohistochemistry assays were carried out as follows: dewaxed sections were incubated in a blocking buffer (PBS containing 5% BSA and 1% Tween-20) for 2 h at environmental temperature, and then with the following primary antibodies diluted in blocking solution (PBS containing 1% BSA and 1% Tween-20): polyclonal anti-TLR4 produced in rabbit (SAB5700684, Sigma-Aldrich, Darmstadt, Germany) (1:200); polyclonal anti-NFκB produced in rabbit (SAB4501989, Sigma-Aldrich) (1:200) overnight at 4 °C. Thus, ascidian sections were incubated with the secondary antibodies (goat anti-rabbit IgG-alkaline phosphatase; A3812, Sigma-Aldrich) diluted 1:50 in blocking buffer (PBS containing 1% BSA and 1% Tween-20) for 90 min at environmental temperature. The slides were washed (washing buffer: PBS containing 1% Tween-20) and stained with the BCIP/NBT chromogen substrate (Sigma-Aldrich). In all experimental control slides, sections were incubated only with the secondary antibodies. Slides were analysed using a light microscope (Leica DM750, Leica Biosystems, Milan, Italy), and images were obtained using an ORMA-Eurotek MDH5 scientific camera (Milan, Italy). The quantification of the immune-positive stained areas in pharynx vessels (percentage of stained cells) on 6 randomly chosen fields (45,000 μm^2^) for each slide was carried out using Image J software (13.0.6).

### 2.3. Extract Preparation and Protein Concentration

After removing the tunic from the specimens (3–5 cm length), the entire bodies were homogenized into polycarbonate tubes with 500 µL of MS buffer under ice and then centrifuged (36,200× *g* for 20 min at 4 °C). The supernatant was collected and the protein concentration was measured according to the Bradford method [42]. The absorbance of ascidian samples was read at 595 nm with MS as a blank, and a calibration curve defined through BSA (bovine serum albumin) was used to protein concentration estimation (mg/mL). Extract concentrations were adjusted to 0.5 mg/mL.

### 2.4. Phenoloxidase (PO)

PO assays were carried out spectrophotometrically according to the Winder and Harris method [43], using L-Dopa (3,4 dihydroxy-L-phenylalanine; Sigma-Aldrich, St. Louis, MO, USA) as a substrate and MBTH (3-methyl-2 benzothiazolinone hydrazone hydrochloride; Sigma-Aldrich, USA) as a specific reagent. Briefly, ascidian extract (50 μL) with trypsin (50 μL) from bovine pancreas (1 mg/mL; Sigma-Aldrich, USA) or distilled water (50 μL; experimental control) was incubated for 20 min at 20 °C in a reaction mixture (50 μL; 5 mM L-DOPA and 20.7 mM MBTH in distilled water). The spectrophotometric absorbance of samples was read within 60 min (5 min intervals) at 505 nm (microplate reader, RAYTO RT-2100C; Guangming New District, China). Enzymatic activities were calculated as units (U) per min, where 1 U = 0.001 ΔA540 min^−1^ mg^−1^ protein.

### 2.5. Glutathione Peroxidase (GPx)

GPx assays were carried out according to the Ross method [44]. Ascidian samples (50 µL) were incubated with TMB (100 µL; 3,3′ 5,5′-tetramethylbenzidine; Sigma-Aldrich, USA) in 96-well flat-bottomed plates. After 30 min of dark incubation, the reaction was stopped with sulfuric acid (H_2_SO_4_) 2 M. The spectrophotometric absorbance of samples was read at 450 nm and the enzymatic activities were expressed in U/mg according to the following equation: U/mg = Abs × Vf/CS (Vf, final volume of the well; CS, sample concentration).

### 2.6. Lysozyme (LYS)

LYS assays were carried out following the Parry method [45]. Briefly, ascidian samples (30 µL) were incubated with a bacterial suspension (270 µL; *Micrococcus lysodeikticus* ATCC 4698, Sigma-Aldrich, USA) in triplicate placed in 96-well flat-bottomed plates. MS buffer (30 µL) was used in the experimental control. The absorbance (450 nm; microplate reader, RAYTO RT-2100C) was measured at 25 °C for 10 min (30 s intervals). An LYS unit was expressed as the amount of sample causing a decrease in absorbance of 0.001/min (U min^−1^), and U/mL was calculated according to the following formula: U/mL = (Δ abs/min^−1^ × dilution factor × 1000)/enzyme volume buffer.

### 2.7. Alkaline Phosphatase (ALP) and Esterase (EST)

For ALP, ascidian extracts were incubated in a 96-well flat-bottomed plate with an equal volume of 4 mM p-nitrophenyl phosphate substrate (Sigma-Aldrich, USA) liquid in 100 mM ammonium bicarbonate containing 1 mM MgCl_2_ (pH 7.8); EST assays were carried out by incubating the same volume of ascidian extract with 0.4 mM p-nitrophenyl myristate substrate (Sigma-Aldrich, USA) in 100 mM ammonium bicarbonate containing 0.5% of Triton X-100 (pH 7.8, 30 °C; Sigma-Aldrich, USA). The kinetics of both enzymes were assessed according to the Ross method [44], spectrophotometrically reading the sample absorbance (microplate reader, RAYTO RT-2100C) for 1 h (5 min intervals) at 405 nm. A unit (U) of ALP and EST activity was expressed as the amount of enzyme required to release 1 µmol of p-nitrophenol produced in 1 min.

### 2.8. Statistical Analyses

To test differences among experimental treatments, a one-way analysis of variance (ANOVA) was performed on the percentage of immune-positive areas and enzymatic activities. When significant differences were found, a pairwise comparison was used to explore differences among experimental groups (Tukey post hoc test). All data analyses were performed using GraphPad software (Prism 10). Values were expressed as the mean ± standard deviation (SD) resulting from three independent experiments. Differences among groups were considered statistically significant for *p* < 0.05.

## 3. Results

### 3.1. TLR4 and NFκB Immunolocalization

**Figure 1:**
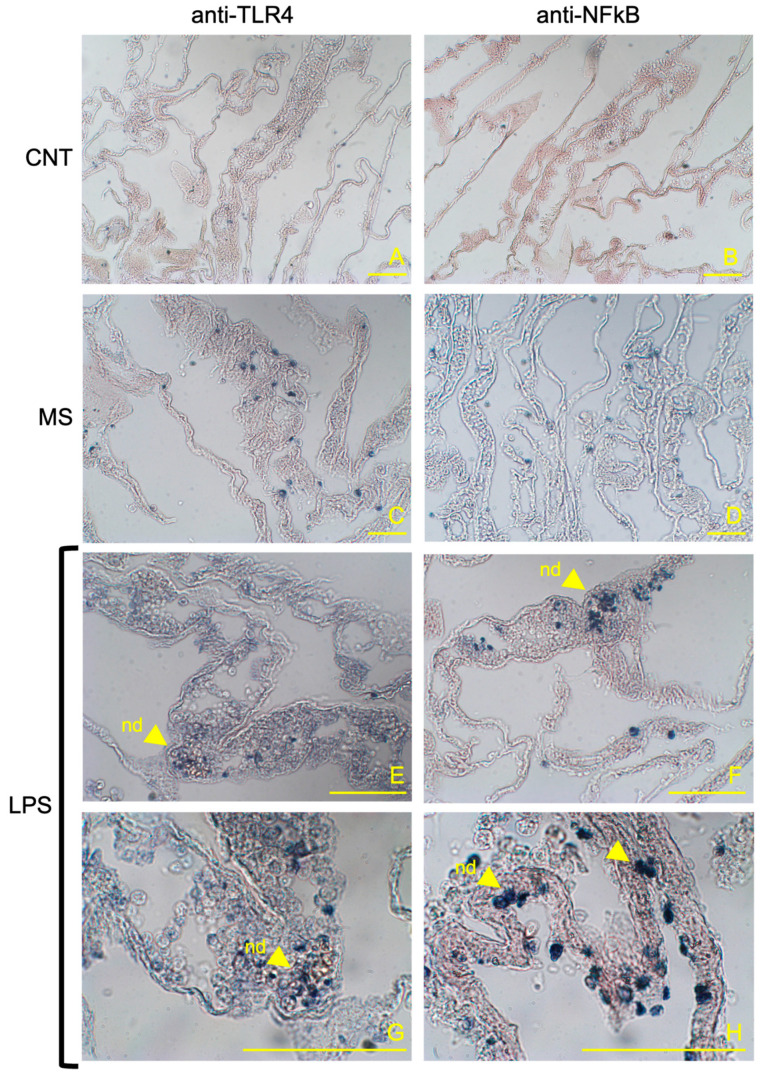
Representative sections of *C. robusta* pharynx at 4 h post-LPS inoculation showing immunohistochemistry with anti-TLR4 and anti-NFκB antibodies. (**A**,**B**) Control ascidians (not injected); (**C**,**D**) sham-injected ascidians inoculated with MS; (**E**,**F**) pharynx vessels at 4 h post-LPS inoculation showing densely populated haemocytes and nodules (nd) marked by the anti-TLR4 and anti-NFκB antibodies, respectively; (**G**,**H**) magnification of marked haemocyte nodules and endothelium (end) in the vessels. Scale bar 50 μm.

**Figure 2:**
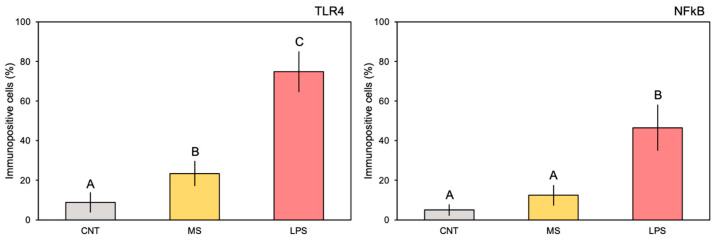
Quantification of the immune-positive stained areas in pharynx vessels (percentage of stained cells; mean values ± SD) from slides belonging to experimental treatments. The letters indicate statistically significant differences (*p* < 0.05) between experimental groups.

The comparison of pharynx slides revealed that very few cells expressed TLR4 (Figure 1A) or NFκB (Figure 1B) markers in the control ascidian pharynx vessels, and some positive cells were observed of MS-inoculated animals (Figure 1C,D). In addition, no positive expression of the examined proteins was observed in the endothelium of control ascidians. Conversely, 4 h after inoculation with *E. coli* LPS, a large number of the vessels were densely populated with haemocytes expressing both proteins (Figure 1E,F). Immuno-positive haemocytes formed numerous large nodules inside the vessel lumen (Figure 1G,H). Positive staining was also observed in the endothelium, indicating the variable expression of TLR4 and, consistently, NFκB markers within this tissue (Figure 1G,H). Quantification of the immune-positive cells (percentage) confirmed significant differences in the staining of both markers; the control and MS-inoculated ascidians showed from 8.8 to 23.4% of TLR4 stained cells and from 5.0 to 12.4% of NFκB stained cells, while significantly higher values were recorded for the LPS-inoculated ascidians compared to the control and MS-inoculated specimens (74.8 and 46.5% of stained cells for TLR4 and NFκB, respectively) (Figure 2; Table 1). No staining was present when the primary antibody was omitted or if pre-immune serum was used.

### 3.2. Enzymatic Response

**Figure 3:**
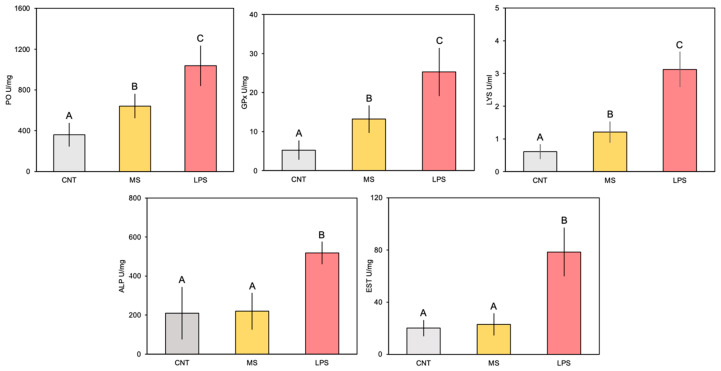
Enzymatic response of phenoloxidase (PO), glutathione peroxidase (GPx), lysozyme (LYS), alkaline phosphatase (ALP) and esterase (EST) in ascidians at 4 h post-inoculation with *E. coli* LPS. The letters indicate statistically significant differences (*p* < 0.05) between experimental groups.

Overall, the enzymatic activities measured in *C. robusta* whole-body extracts underwent a significant increase in ascidians 4 h after LPS inoculation (Figure 3; Table 2). In detail, PO activity increased in animals belonging to the MS and LPS groups with respect to controls, and even more so in specimens injected with *E. coli* (Figure 3). However, multiple comparison analysis showed significantly higher values only for ascidians injected with LPS compared to controls and MS-injected organisms (Figure 3; Table 2). The GPx trend among experimental groups was similar to PO (Figure 3), with significantly higher values for the LPS-injected animals (Table 2). Regarding LYS, the one-way ANOVA evidenced significant differences between treatments (Table 2). Overall, activity was higher in the injected animals than in the control group (Figure 3). The highest LYS activity was detected in the LPS group. Also in this case, Tukey’s test showed significant differences for ascidians injected with *E. coli*. Elevated ALP and EST activity was observed, with values 2-fold greater than in control and MS-injected animals (Figure 3). Statistical analysis revealed highly significant differences only for animals challenged with LPS compared to the other treatments, for both enzymes (Figure 3; Table 2).

## 4. Discussion

This study confirms that the innate immune signalling pathway activated by LPS in *C. robusta* is evolutionarily conserved and involves TLR-NFκB activities, in agreement with previous observations that highlighted the activation of this key immune pathway against invading pathogens and other potential threats to an ascidian host [6,7]. This is not surprising, given their key phylogenetic position in chordate evolution, generally considered a sister group of vertebrates [1,2,46,47]. In our *C. robusta* model, the NFκB signalling pathway appears to have been activated in pharynx vessels as a defence response against the bacterial LPS stimuli through the involvement of TLR receptors. The upregulation of TLR4 was found at 4 h post-inoculation, when TLR4-producing haemocytes densely populated the lumen of the pharynx vessels. In addition, numerous nodules were formed in the vessels by TLR4-producing haemocytes, giving a distinctive inflammatory signature to the vessels. Consistently, an increase in circulating haemocytes expressing the key immune protein NFκB was also shown 4 h after LPS challenge, indicating the probable activation of a TLR-NFκB-dependent pathway.

Nodules in the ascidian vessels were made up of tightly packed cells and were often connected or closely associated with the internal part of the endothelium. These haemocytes, containing TLR4 and NFκB transcripts in membranes and nuclei/cytoplasm, respectively, could be retained as activated cells engaged in inflammatory response in both the pharynx and haemolymph. The vascular endothelium was also involved in the response; although no continuous staining was observed in the endothelial tissue, several cells expressed the two proteins. Since several cells were shown to be maintained as proliferating cells, endothelium-associated haematopoietic nodules could develop following LPS stimulation [48,49,50,51]. In fact, there is the possibility that nodular stem cells differentiate into cell lines that circulate in the haemolymph and are recruited to inflamed sites [5,48]. Our results showed that the pharyngeal tissues of *C. robusta* can be stimulated by an LPS response and that they participate in immunity through vascular endothelium and nodules potentially acting as inflammatory haemocytes. This ascidian LPS-induced inflammatory response was also supported by the lack of pharynx inflammation observed in sham-injected animals inoculated with MS.

Overall, *C. robusta* tissue extracts showed a marked and significant upregulation of enzymatic activity following LPS injection. PO activity 4 h post-LPS injection was approximately two-fold higher compared to untreated ascidians. After PAMP recognition and subsequent activation, the PO-cascade hydroxylate monophenol and diphenol substrates in melanin polymeric deposits produced highly cytotoxic defences and barriers against foreign cells or molecules [4,23,24,52]. Comparable PO up-activation has already been documented in the haemocytes, body wall and tunic extracts of *C. robusta* model species, showing its involvement in inflammatory responses following LPS challenge [23,24,52]. Based on their biochemical properties, several POs have been described among ascidian species, presumably related to various functional roles [4,23]. Although they show catecholate activity, differences in size, trypsin sensitivity, activating substances, and SDS chain sensitivity have been found between species [4,23]. Additionally, differences within the same species have been found, for example, in terms of different sizes and trypsin enzyme sensitivity in granular haemocytes and morula cells [23,29]. Concurrent with the activation of the cytotoxic PO system, there was corresponding heightened antioxidant activity of GPx, an enzyme that generally scavenges hydrogen peroxide [53]. The significant values (greater than four-fold) manifested 4 h post-LPS injection in challenged specimens suggest this enzyme’s involvement in ascidian inflammatory response. This is consistent with the induction of oxidative stress conditions during the invertebrate immune response under LPS stimuli, as a result of the oxidative bursts and as a product of the PO system’s activity [30,54].

LYS exhibited clear and significant up-regulated activity in *C. robusta* extracts 4 h post-LPS challenge, three-fold greater than untreated animals. These findings further corroborate the involvement of LYS in innate immune defence and in the bacterial intracellular digestion of this invertebrate that feeds by filtering seawater and which is often exposed to high concentrations of microorganisms [33]. For example, previous observations from our group showed that the spatial mRNA expression of g-type lysozymes in adult specimens of *C. robusta* was detected mainly in pharynx, stomach and intestine tissues from 1 to 4 h after LPS injection [33], which is consistent with the hypothesis that LYS is expressed predominantly in organ tissues exposed to the external environment or in haematopoietic tissues [55,56]. We also analysed ALP and EST activities 4 h post-LPS challenge. The results of the assays conducted on *C. robusta* wall-body extracts suggested a correlation between the inflammatory response and the modification of these enzymatic parameters. The involvement of ALP and EST in the innate immune response to LPS had already been observed in other marine invertebrates, such as nematodes and molluscs [34,35], as well as being considered among the most interesting markers during regenerative inflammatory processes [57].

## 5. Conclusions

In conclusion, our results provide convincing evidence of the involvement of the entire pharynx in the inflammatory response of *C. robusta*, manifesting 4 h after LPS challenge, confirming the stimulation of the TLR-NFκB-dependent pathway against pathogenic agents. Also, to our knowledge, this is the first study to provide functional indications regarding the activities of several enzymatic parameters in the innate immune response to LPS injection in wall-body extracts of ascidian animal models. To fill the knowledge gaps regarding the hierarchically organized set of molecular, cellular and organismal networks involved in immune interactions with pathogens and the subsequent evolution of immune responses, it is essential to implement new studies that use such a broad-based approach.

## Figures and Tables

**Table 1:** Summary of the one-way ANOVAs carried out on marker expression (TLR4 and NFκB) among experimental treatments on immune-positive stained areas in pharynx vessels (percentage of stained cells).

Ordinary One-Way ANOVA	F	*p* Value	R Square
TLR4	170.00	<0.0001	0.95
NFκB	51.24	<0.0001	0.87

**Table 2:** Summary of the ordinary one-way ANOVAs carried out on enzymatic activities among experimental groups.

Ordinary One-Way ANOVA	F	*p* Value	R Square
PO	15.29	0.0044	0.83
GPx	16.08	0.0039	0.84
LYS	30.52	0.0007	0.91
EST	42.11	<0.0001	0.84
ALP	12.09	0.0028	0.72

## Data Availability

The data presented in this study are available on request from the corresponding author.

## Supplementary Materials

The following supporting information can be downloaded at: https://www.mdpi.com/article/10.3390/ani14152169/s1: Figure S1: Sequence alignments; Table S1: Sequence accession numbers.
