# Supplementary material for: The Interplay of TLR-NFκB Signalling Pathway and Functional Immune-Related Enzymes in the Inflammatory Response of Ciona robusta
Source: Animals (Basel). 2024 Jul 25;14(15):2169. doi: 10.3390/ani14152169 (PMC11310991; doi:10.3390/ani14152169)
Supplement: Supplementary file 1 [file animals-14-02169-s001.zip › animals-3093891-supplementary.pdf]

Supplementary Materials

The Interplay of TLR-NFκB Signalling Pathway and Functional Immune-Related Enzymes in the Inflammatory Response of *Ciona robusta*

Luca Bisanti [1,2], Claudia La Corte [1,2], Mariano Dara [1,2], Federica Bertini [1,2], Jacopo Vizioli [3], Maria Giovanna Parisi [1,2], Matteo Cammarata [1,2,*] and Daniela Parrinello [1,2]

1 Marine Immunobiology Laboratory, Department of Earth and Marine Sciences, University of Palermo, 90128 Palermo, Italy; luca.bisanti@unipa.it (L.B.); claudia.lacorte@unipa.it (C.L.C.); mariano.dara@unipa.it (M.D.); federica.bertini02@unipa.it (F.B.); mariagiovanna.parisi@unipa.it (M.G.P.); daniela.parrinello@unipa.it (D.P.)

2 National Biodiversity Future Center (NBFC), 90133 Palermo, Italy

3 Laboratoire Protéomique, Réponse Inflammatoire et Spectrométrie de Masse (Inserm U1192), Département de Biologie, Université de Lille, F-59000 Lille, France; jacopo.vizioli@univ-lille.fr

* Correspondence: matteo.cammarata@unipa.it

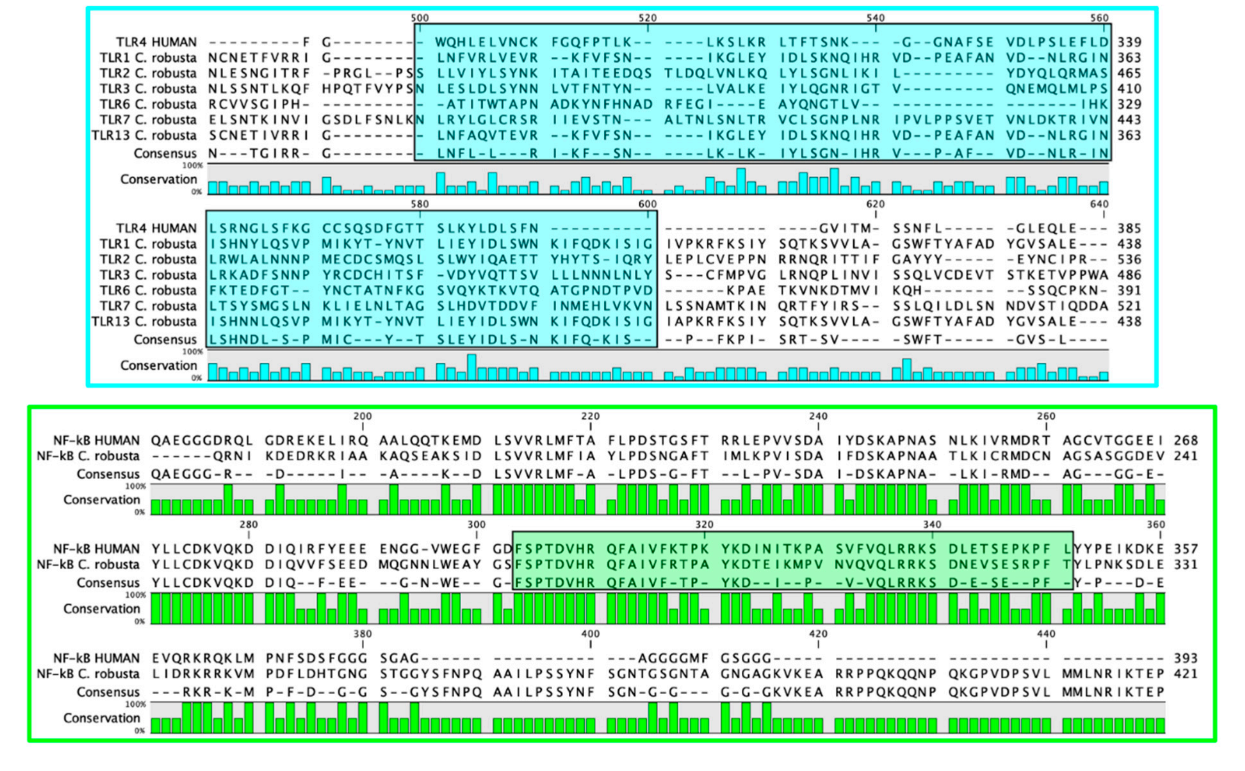

Figure S1. The sequences of human TLR4 and NFκB used for alignment with *C. robusta* deposited sequences for systematic and comprehensive identification of antigen binding sites. The consensus contained in the coloured box improves the understanding of antigenic interactions that determine cross-reactions in the immunoblotting assays performed using *C. robusta* wall-body extracts. The references and accession numbers of the sequences used in the alignment are reported in supplementary Table S1.

Species	Protein	Accession number
Homo sapiens	Toll-like Receptor 4 (TLR4)	UniProtKB O00206
Ciona robusta	Toll-like Receptor 1 (TLR1)	GenBank NP_001159599.2
Ciona robusta	Toll-like Receptor 2 (TLR2)	GenBank NP_001159600.1
Ciona robusta	Toll-like Receptor 3 (TLR3)	GenBank XP_002120237.1
Ciona robusta	Toll-like Receptor 6 (TLR6)	GenBank XP_002130253.1
Ciona robusta	Toll-like Receptor 7 (TLR7)	GenBank NP_001231972.1
Ciona robusta	Toll-like Receptor 13 (TLR13)	GenBank XP_002120520.2
Homo sapiens	Nuclear Factor kappa B (NFκB)	UniProtKB P19838
Ciona robusta	Nuclear Factor kappa B (NFκB)	GenBank NP_001071772.1

Table S1. References and accession numbers of the sequences used for the sequence alignment of *C. robusta* with the antigenic epitopes of the antibodies employed in the immunoblotting assay.
